# Burnout and engagement among PhD students in medicine: the BEeP study

**DOI:** 10.1007/s40037-020-00637-6

**Published:** 2020-12-07

**Authors:** Rashmi A. Kusurkar, Stéphanie M. E. van der Burgt, Ulviye Isik, Marianne Mak-van der Vossen, Janneke Wilschut, Anouk Wouters, Andries S. Koster

**Affiliations:** 1 Amsterdam UMC, Research in Education, Faculty of Medicine, Vrije Universiteit, Amsterdam, The Netherlands; 2 LEARN! Research Institute for Learning and Education, Faculty of Psychology and Education, VU University Amsterdam, Amsterdam, The Netherlands; 3 Center for Evidence Based Education, location AMC, Amsterdam, The Netherlands; 4 Dutch Institute of Clinical Auditing, Leiden, The Netherlands; 5 Department of Pharmaceutical Sciences, Utrecht University, Utrecht, The Netherlands

**Keywords:** Burnout, Motivation, Engagement, PhD students, Medicine

## Abstract

**Introduction:**

Using a self-determination theory framework, we investigated burnout and engagement among PhD students in medicine, and their association with motivation, work-life balance and satisfaction or frustration of their basic psychological needs.

**Method:**

This cross-sectional study was conducted among PhD students at a university medical centre (*n* = 990) using an electronic survey on background characteristics and validated burnout, engagement, motivation and basic psychological needs questionnaires. Cluster analysis was performed on the burnout subscale scores to find subgroups within the sample which had similar profiles on burnout. Structural equation modelling was conducted on a hypothesized model of frustration of basic psychological needs and burnout.

**Results:**

The response rate was 47% (*n* = 464). We found three clusters/subgroups which were composed of PhD students with similar burnout profiles within the cluster and different profiles between the clusters. Cluster 1 (*n* = 199, 47%) had low scores on burnout. Clusters 2 (*n* = 168, 40%) and 3 (*n* = 55, 13%) had moderate and high burnout scores, respectively, and were associated with low engagement scores. Cluster 3, with the highest burnout scores, was associated with the lowest motivational, engagement, needs satisfaction and work-life balance scores. We found a good fit for the “basic psychological needs frustration associated with burnout” model.

**Discussion:**

The most important variables for burnout among PhD students in medicine were lack of sleep and frustration of the basic psychological needs of autonomy, competence and relatedness. These add to the factors found in the literature.

**Electronic supplementary material:**

The online version of this article (10.1007/s40037-020-00637-6) contains supplementary material, which is available to authorized users.

## Introduction

Burnout has been identified as a global problem among medical students, residents and physicians, and is on the rise [1–4]. The percentage of physicians having at least one symptom of burnout in the US increased from 45% to 54% between 2011 and 2014 [5]. “PhD students in medicine” as a group has been neglected in burnout research. The reason PhD students in medicine (with or without a clinical background) deserve to be treated as a separate group from other PhD students is because this group works in a hospital or clinical setting, may be supervised by physicians with PhDs and may experience elements of the medical culture and hierarchy in ways that may contribute to burnout [4]. Thus the context is very different from a general university setting. Within PhD students in medicine, PhD students with clinical backgrounds have been reported to have different motivation, financial position and confidence as professionals than those with other scientific backgrounds [6]. PhD students actively working with patients are also expected to have more workload and conflict in balancing clinical or patient responsibilities along with their PhD research work. The current study therefore aimed to explore burnout and the factors influencing burnout among PhD students in medicine.

“Job burnout is a psychological syndrome that involves a prolonged response to chronic interpersonal stressors on the job” [7]. The professional consequences of burnout are lower productivity at work, unprofessionalism, increased errors and higher chances of quitting the field of work. In case of health professionals (which would include PhD students in medicine with patient responsibilities), burnout can lead to consequences such as dissatisfied patients, lower patient safety, higher patient mortality and higher cost of inefficiently delivered healthcare [8].

In this study “PhD students in medicine” are students at a university medical centre who have completed their Master’s degree and are undergoing residency/specialization training, or are completing PhD research concurrently with their residency training or professional practice, or have completed a Bachelor’s degree in Medicine and are following an MD-PhD program, or non-medical and/or non-clinical graduates completing their research in a non-clinical or a clinical department.

There are several studies on satisfaction of PhD students, stress and depression, and well-being, but the findings of PhD students in medicine have not been reported separately [9–11]. In a review of the factors influencing PhD students’ well-being, achievement and PhD completion, it is clear that: a) PhD students in medicine have not been identified as a separate group; b) their findings are not reported separately; c) more factors influencing success in PhD have been investigated than well-being; d) only one of the studies focuses on burnout; and e) no specific theoretical framework has been used for studying burnout and engagement [11]. (See Table A1 in the Electronic Supplementary Material).

Burnout is described as having three dimensions: exhaustion, cynicism and perceived negative efficacy [7]. Exhaustion means feeling physically and emotionally exhausted, cynicism involves feelings of detachment from one’s work, and perceived negative efficacy involves a feeling of incompetence in work. Generally, cynicism appears first (it also has the highest score), followed by exhaustion; negative personal efficacy may even be absent [12]. Since one of our research goals was to provide recommendations for supporting these PhD students, we explored not only burnout and factors influencing it, but also engagement in work and motivation (which is an important driver of performance) and the factors enhancing them. Engagement is defined as “a positive, fulfilling, and work-related state of mind that is characterized by vigour, dedication and absorption” [13]. We used the framework of Self-Determination Theory (SDT) to investigate this phenomenon because it provides a basis for investigating burnout and engagement through frustration or satisfaction of basic psychological needs, respectively [14]. Thus, this study aimed to investigate burnout and engagement among PhD students in medicine, and how these variables are associated with the quality of their academic motivation [14], work-life balance [15], quality of sleep [16], perceived conflict in work-related responsibilities and satisfaction or frustration of their basic psychological needs [14]. (See Table A2 in the Electronic Supplementary Material for descriptions of these variables).

### Self-determination theory framework

Self-determination theory (SDT) [14, 17] is a macro-theory of human motivation, which puts the fulfilment of three basic psychological needs—autonomy (feeling of choice), competence (feeling of capability) and relatedness (feeling of belonging)—at the epicentre of an individual being autonomously motivated for learning or work, his/her well-being, happiness, creativity and performance. This theory considers the quality of motivation (the why of motivation) more important than the quantity (how much) and describes the quality of motivation as autonomous or controlled. Autonomous motivation is derived out of genuine interest and/or great personal value for learning or work [18]. Controlled motivation stands for persuasion of learning or work because of internal or external pressure or in the expectation of a reward [18]. SDT advocates that the more autonomous the motivation, the better the observed outcomes, namely: deep learning, high academic or work performance, better adjustment and positive well-being [14, 17]. Satisfaction of the basic psychological needs can move a student from controlled towards autonomous motivation and is also directly associated with engagement. On the contrary, frustration of these needs can move a student from autonomous towards controlled motivation and is also directly associated with burnout.

**Fig. 1:**
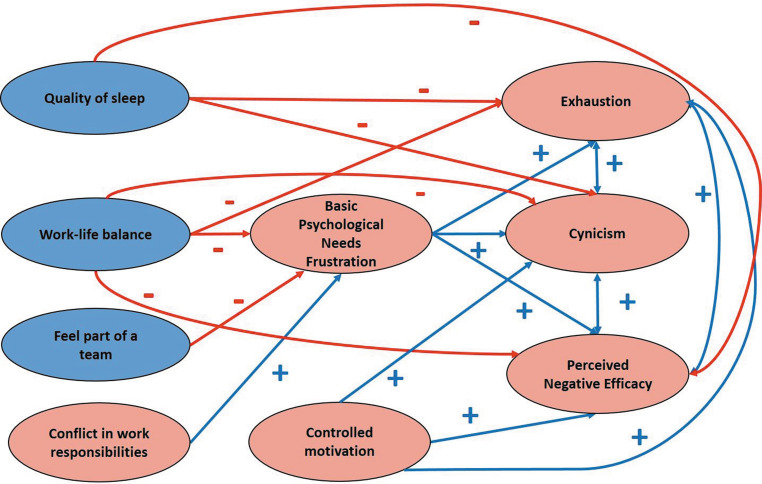
Hypothesized model for basic psychological needs frustration and burnout

Our hypothesized model on basic psychological needs frustration-burnout is depicted in Fig. 1.

Our research questions for this study were:

1. How do PhD students in medicine score on burnout, work engagement, quality of motivation for PhD, work-life balance, conflict in work responsibilities, feeling part of a team and quality of sleep? Can we find patterns in how they score on the burnout subscales?
2. Are there differences in the burnout scores of males and females, clinical and non-clinical departments, and working with patients, in a lab or in an office?
3. How do PhD students in medicine score on the satisfaction or frustration of their basic psychological needs of autonomy, competence and relatedness?
4. How are burnout, quality of motivation for PhD, work-life balance, quality of sleep, conflict in work responsibilities, feeling part of a team, frustration of their basic psychological needs and background variables associated with each other?

## Methods

### Setting

This cross-sectional study was conducted at a University Medical Centre in Amsterdam, the Netherlands. PhD training in the Netherlands is generally a 4-year program with training in research skills and other non-cognitive skills (such as presenting in English) interspersed between data collection, analysis, interpretation and publication of about four empirical studies which form the PhD thesis. Thus the PhD is not divided into coursework and thesis phases as in some other parts of the world [19]. Also PhD students are appointed as employees and receive a salary instead of a stipend. Some PhD students who have a medical degree combine their PhD work with their residency training, while others pursue one at a time. Some PhD students have non-medical backgrounds even though they may be pursuing a PhD in a medical department. All PhD students in medicine registered at the different research institutes in this medical centre (*n* = 990) were invited to fill out an electronic survey using Net Questionnaire. The data were collected from September to November 2018 and two reminders were sent. We obtained ethical approval from the Netherlands Association for Medical Education—Ethical Review Board (Folder no. 2018.5.13).

### Background variables

We collected anonymous data after written informed consent, participation was voluntary and no incentives were provided. See Tables A2 and A3 in the Electronic Supplementary Material for the details of the survey and demographic characteristics of the participants, respectively.

### Data analyses

Descriptive statistics and Pearson’s correlations for all included variables were computed (research questions 1 and 3). We tested for differences (research question 2) in the variable scores for gender, clinical versus non-clinical departments and work setting using students unpaired t‑tests or ANOVAs, as appropriate. To answer the second part of our research question 1, we performed K‑means cluster analysis using the three burnout subscale scores. We did this because the MBI-SS [13] does not provide any cut-off scores for classifying burnout as “high”, “moderate” or “low”. Cluster analysis helped us to group PhD students who had similar scores on the three subscales of burnout with each other [20]. We validated the cluster solution using the random half-splitting method and computing the Cohen’s kappa as a measure of cluster stability. Clustering effectively reduced the within-groups variability of burnout scores by more than 50%, compared with the score variability before clustering. Therefore, clustering was considered effective. Clustering also allowed us to compare the groups with each other for their scores on the dependent variables using multiple analysis of variance (multiple ANOVAs), followed by comparison of group means using Bonferroni adjustments. Cohen’s d was used to characterize the effect size for differences between individual means, whenever statistically significant [21].

To answer our research question 4, we conducted Structural Equation Modelling analysis using AMOS 18 for testing the hypothesized model depicted in Fig. 1 [22, 23]. The indices used for estimating goodness of fit of the model were: Root Mean Square Error of Approximation (RMSEA <0.05), Comparison of Fit Index (CFI >0.95), Tucker Lewis Index (TLI >0.95), and Standardized Root Mean Square Residual (SRMR <0.05) [22, 23].

## Results

The response rate was 47% (*n* = 464). The majority were 25–34 years old, Dutch females (*n* = 371, 80%), married/in a relationship, and childless. Thirty-three percent had a medicine-related degree, 52% were from clinical departments, 56% worked in an office, 27% in a lab and 17% with patients; 68% worked beyond their contract hours. The demographic characteristics and the Pearson’s correlations between all study variables are depicted in Tables A3 and A4 in the Electronic Supplementary Material.

A limited number of statistically significant differences (*p* < 0.05) between genders, departments, or work settings were found. Concerning gender, we found that work-life balance was significantly better (males: 1.92 ± 0.62, females: 2.07 ± 0.57, *p* = 0.038, *d* = 0.26) and vigour was significantly poorer (males: 2.22 ± 0.74, females: 1.96 ± 0.74, *p* = 0.004, *d* = 0.35) in females as compared with males. Concerning clinical *versus* non-clinical departments, autonomy frustration was significantly higher (clinical: 3.89 ± 1.21, non-clinical: 3.58 ± 1.20, *p* = 0.013, *d* = 0.26) and conflict between work responsibilities was significantly higher (clinical: 5.10 ± 2.59, non-clinical: 4.22 ± 2.32, *p* < 0.001, *d* = 0.36) among PhD students from clinical departments. Concerning work setting, work-life balance was significantly better among PhD students who worked in an office (2.16 ± 0.60) as compared to those who worked in a lab (1.89 ± 0.54) or with patients (1.90 ± 0.56): *p* < 0.001 in both comparisons (*d* = 0.48 and 0.45, respectively). Conflicts with work responsibilities were significantly higher among PhD students who worked with patients (6.14 ± 2.28) than those who worked in an office (4.61 ± 2.50) or a lab (3.99 ± 2.24): *p* < 0.001 in both comparisons (*d* *=* 0.64 and 0.90, respectively).

When we tried to classify PhD students, based on their burnout scores, three clusters were found with increasing scores on the subscales exhaustion, cynicism and perceived negative efficacy (Tab. 1).

**Table 1:** Cluster analysis on the basis of scores on the burnout subscales (range 1–6)

Cluster	*N* (%)	Exhaustion (mean ± SD)	Cynicism (mean ± SD)	Perceived negative efficacy (mean ± SD)
1 Low scores on burnout	199 (47%)	1.65 **±** 0.73	1.45 **±** 0.86	1.69 **±** 0.67
2 Medium scores on burnout	168 (40%)	3.34 **±** 0.82	3.41 **±** 1.00	2.19 **±** 0.52
3 High scores on burnout	55 (13%)	3.69 **±** 0.94	4.59 **±** 0.85	3.62 **±** 0.61

Cohen’s kappa for cluster stability = 0.95 (> 0.8 is considered good)

Cluster 1 had low scores on burnout. Clusters 2 and 3 with medium and high scores on burnout were associated with low engagement scores. Cluster 3, with high burnout scores, was associated with the lowest autonomous motivation, engagement, needs satisfaction, perception of being part of a team, and feeling refreshed in the morning and the highest controlled motivation, needs frustration and conflict in work responsibilities (Tab. 2). Effect sizes for the differences between clusters 1 and 2, and between clusters 2 and 3, of the engagement and basic psychological needs scores were substantial (*d* > 0.8 in many cases); relatedness scores were relatively less affected (see Tab. 2, and Figure A1 in the Electronic Supplementary Material).

**Table 2:** Comparison of dependent variable scores between clusters (Multiple ANOVAs)

Variable (range of scores)	Cluster 1 Low burnout scores	Cluster 2 Medium burnout scores	Cluster 3 High burnout scores	Statistical significance of ANOVA	Difference between Mean 1 and Mean 2	Difference between Mean 2 and Mean 3
	*Mean1* *±* *SD* *(n* *=* *199)*	*Mean2* *±* *SD* *(n* *=* *168)*	*Mean3* *±* *SD* *(n* *=* *55)*	*F, η*^*2*^ *significance*	*Effect size* *(d)*	*Effect size* *(d)*
Autonomous motivation(1–5)	4.17_a_ ± 0.38	3.90_b_ ± 0.42	3.31_c_ ± 0.83	68.6, 0.256***	0.56	1.21
Controlled motivation (1–5)	1.79_a_ ± 0.53	1.97_b_ ± 0.62	2.37_c_ ± 0.74	37.4, 0.159***	0.31	0.69
Engagement (0–4)						
Vigor (0–4)	2.42_a_ ± 0.59	1.82_b_ ± 0.61	1.13_c_ ± 0.61	108.8, 0.372***	1.00	1.14
Dedication (0–4)	2.93_a_ ± 0.45	2.42_b_ ± 0.55	1.74_c_ ± 0.74	114.1, 0.363***	0.95	1.27
Absorption (0–4)	2.52_a_ ± 0.64	2.26_b_ ± 0.58	1.56_c_ ± 0.76	46.7, 0.196***	0.41	1.10
Satisfaction of BPN (1–7)	5.20_a_ ± 0.65	4.47_b_ ± 0.66	3.82_c_ ± 0.85	103.7, 0.361***	1.06	0.95
Autonomy satisfaction (1–7)	4.96_a_ ± 0.79	3.91_b_ ± 0.93	3.36_c_ ± 1.08	97.3, 0.346***	1.17	0.62
Competence satisfaction (1–7)	5.27_a_ ± 0.79	4.66_b_ ± 0.84	3.55_c_ ± 1.15	87.1, 0.318***	0.71	1.29
Relatedness satisfaction (1–7)	5.36_a_ ± 1.03	4.83_b_ ± 1.10	4.54_b_ ± 1.36	16.3, 0.082***	0.48	n. s.
Frustration of BPN (1–7)	2.60_a_ ± 0.77	3.55_b_ ± 0.71	4.24_c_ ± 0.83	130.1, 0.429***	1.26	0.92
Autonomy frustration (1–7)	2.98_a_ ± 0.96	4.27_b_ ± 0.95	4.74_c_ ± 1.06	109.6, 0.377***	1.32	0.48
Competence frustration (1–7)	2.72 _a_ ± 1.05	3.62_b_ ± 1.16	4.95_c_ ± 1.23	90.7, 0.347***	0.81	1.19
Relatedness frustration (1–7)	2.11_a_ ± 1.07	2.75_b_ ± 1.17	3.04_b_ ± 1.39	21.0, 0.106***	0.56	n. s.
Work-life balance (1–3)	2.24_a_ ± 0.56	1.82_b_ ± 0. 55	1.93_b_ ± 0.55	28.6, 0.121***	0.76	n. s.
Conflict at work (1–10)	3.91_a_ ± 2.27	5.51_b_ ± 2.40	5.00_b_ ± 2.66	20.7, 0.087***	0.68	n.s
Belong to team (1–6)	4.20_a_ ± 1.40	3.41_b_ ± 1.50	2.61_c_ ± 1.45	31.4, 0.128***	0.55	0.56
Quality of sleep (0–10)	7.41_a_ ± 1.53	6.48_b_ ± 1.85	6.59_b_ ± 1.71	16.2, 0.081***	0.55	n. s.
Feeling refreshed (1–5)	3.60_a_ ± 0.81	2.85_b_ ± 0.91	2.61_c_ ± 0.98	40.6, 0.170***	0.85	0.27

Significance of the ANOVA analyses is indicated by the test value of the between clusters versus within clusters mean square (F) and the overall effect size, expressed as fraction of explained variance (η2). Cluster means with different subscripts differ statistically significant from each other (*p* < 0.01, Bonferroni post-hoc test). Effect sizes for the difference between cluster means (Cohen’s d) were calculated from the difference in means and the pooled standard deviation, derived from the ANOVA analyses

***p < 0.001 in all cases; n. s., not significantly different. The means which have different subscripts differ from each other significantly. The means with the same subscript do not differ significantly. *BPN* basic psychological needs.

**Fig. 2:**
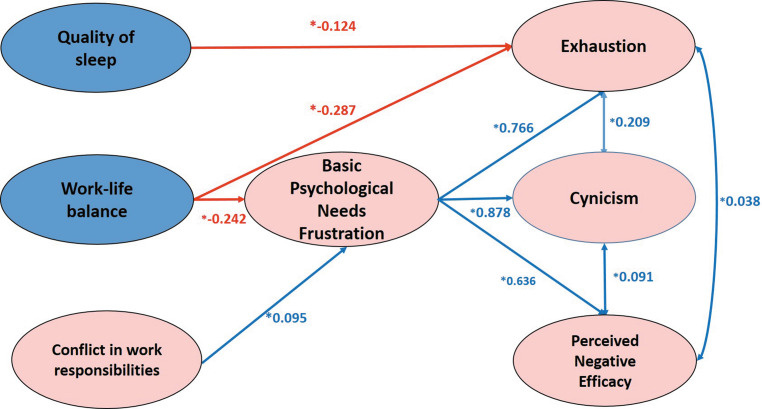
Structural equation model of BPN frustration and burnout. Indirect effects: Work Life Balance-Basic Psychological Needs Frustration-Exhaustion (*−0.185); Work Life Balance-Basic Psychological Needs Frustration-Cynicism (*−0.213); Work Life Balance-Basic Psychological Needs Frustration-Perceived Negative Efficacy (*−0.154)

Relationships between basic psychological needs and burnout scores for the sampled PhD students as a whole were investigated using structural equation modelling. We did not find a good fit for the hypothesized model (Fig. 1). We therefore removed all the non-significant relationships from the model one by one and finally arrived at the model depicted in Fig. 2, which had a good fit with our data, RMSEA = 0.044 (<0.06), CFI = 0.986 (>0.95), TLI = 0.976 (>0.95), SRMR = 0.041 (<0.05). Quality of sleep was negatively associated with exhaustion. Work-life balance was negatively associated with basic psychological needs frustration, directly and indirectly with exhaustion, and indirectly with cynicism and perceived negative efficacy. Conflict in work responsibilities was negatively associated only with basic psychological needs frustration, and did not have any direct or indirect effects on the burnout subscale scores. Basic psychological needs frustration was associated with exhaustion, cynicism and perceived negative efficacy.

## Discussion

As hypothesized, we found that PhD students from clinical departments had a poorer work-life balance and higher conflict between work responsibilities. They also had higher autonomy frustration, which can be expected due to the conflict. A previous study has investigated differences between the motivation and expectations of PhD students with clinical versus science backgrounds [6]. PhD students with clinical backgrounds were generally older, and found lab work difficult. Although they started their PhD training perceiving themselves as professionals, they were treated by their departments as students. They perceived this as frustrating [6]. But this study did not investigate burnout among these students [6]. PhD students working with patients had a poorer work-life balance and higher conflict with work-related responsibilities than those working in a lab or an office. Clinical residents have been reported to have higher burnout scores in an earlier study owing to high patient load, long working hours and low autonomy [1]. A national study on Dutch residents has reported a high percentage (21%) of burnout [3]. If PhD work is conducted on top of these circumstances, much worse outcomes can be expected.

We also found three groups based on PhD students’ scores on the burnout subscales: *Low, moderate and high*. Contrary to the literature, we did not find gender differences between the three burnout subscale scores [24]. The “high” group had the worst outcomes for engagement and motivation, and basic psychological needs satisfaction and frustration. This finding differs from the study on Dutch residents, in which the authors found that high burnout could be associated with high engagement or low engagement [3].

We were able to find evidence for a modified model of the relationship between basic psychological needs frustration and burnout than our hypothesized model. Sleep and basic psychological needs frustration have important effects on burnout, while work-life imbalance and conflict in work responsibilities have an important positive effect on basic psychological needs frustration. In addition, work-life balance has an important indirect negative effect on burnout through its effect on basic psychological needs frustration. These findings about basic psychological needs frustration and burnout add to the literature on this topic. Similar results were found in an earlier study among pharmacists, in which basic psychological needs frustration was associated with low vitality [25]. We could not find evidence for a hypothesized positive relationship between basic psychological needs satisfaction and engagement using structural equation modelling. A similar lack of evidence for a positive relationship between basic psychological needs satisfaction and vitality has been reported earlier by Tjin A Tsoi et al. [25] We suggest that preventing frustration of basic psychological needs is more important for preventing burnout than ensuring satisfaction of basic psychological needs [25]. This could be due to the relatively high autonomous motivation for pursuing a PhD project in the sampled population. It can be expected that their perception of autonomy and competence is not so easily changed by external influences. On the other hand, frustration of autonomy and competence by conflicting work requirements and/or inadequate support and guidance can easily lead to a sense of frustration and burnout.

### Practical applications/recommendations

Using a cluster analysis, which is a person-centred research analysis [26], for creating groups made of similar characteristics on burnout helped us propose customized recommendations for these different groups. General recommendations, based on Self-Determination Theory, for PhD students, supervisors and organizations, related to the prevention of frustration and support of satisfaction of autonomy, competence and relatedness are summarized in Table A5 in the Electronic Supplementary Material.

### Specific recommendations for the three clusters

The low burnout scores cluster seems to have favourable scores on all factors except the three subscales of engagement: vigour, dedication and absorption. We recommend training for the students in this cluster on how to become more engaged in their PhD work. The students in the moderate burnout scores cluster seem to have unfavourable scores on autonomy satisfaction and frustration, engagement—vigour, work-life balance, conflict in work responsibilities, feeling of belongingness to a team and feeling refreshed on waking up. For students in this cluster, we recommend that the supervision team engages in discussion with their students about how to maintain autonomy in work, about reducing the conflict in work responsibilities, and perpetuating team spirit, while the student gets help with organizing his/her schedule, work-life balance and sleep. The high burnout cluster students seem to have low autonomous motivation, very low engagement, low autonomy and competence satisfaction, high autonomy frustration, poor feelings of belongingness to a team, conflict in work responsibilities and do not feel refreshed on waking up. We recommend that research institutes and human resources departments provide training for the students in this cluster on structuring their work, personal and leisure activities, and resolution of problems related to poor supervision and basic psychological needs frustration.

### Further research questions

Which other variables are important for burnout and engagement among PhD students in medicine? Can our results be replicated in other countries in similar contexts? In addition, we think that in-depth qualitative research to get more detailed information about the stressors and energizers experienced by students in their PhD work would add to the existing literature.

### Limitations

Our study has several limitations. First of all we used self-report measures, which does not give an indication of actual burnout among the PhD students. But this is true for most burnout studies in the literature, and in spite of this we think this study adds important insights to the literature. The cross-sectional design is also a limitation and a longitudinal design would definitely benefit such research. We had a response rate of 47%, which could have created a response bias. We believe this low response rate to be random as we collected data anonymously. In spite of a relatively low response rate, we believe that our results add to the literature on burnout and engagement among PhD students in medicine. We wanted to investigate PhD students particularly with clinical responsibilities, but our sample contained only a small percentage (17%) of such students. We did find evidence that clinical responsibilities can interfere with PhD work. We recommend a similar study with a bigger sample size of PhD students with clinical duties to further explore the differences. Also, this study was conducted at a single medical centre. We recommend multicentre studies in the future in the interest of generalizability. We could have missed important variables influencing burnout and engagement outside of SDT, as we collected data and conducted the analysis using the variables included in the SDT framework. But we expect to have covered all the variables important from the SDT perspective and thus have a strong theoretical foundation for our work. In future studies, more variables beyond the ones in our study could be included.

## Conclusion

The most important variables, found in this study, for burnout among PhD students in medicine students are lack of sleep and frustration of the basic psychological needs of autonomy, competence and relatedness. Work-life imbalance and conflict in work responsibilities are associated with basic psychological needs frustration. The model of basic psychological needs frustration being associated with burnout adds to the literature.

## Caption Electronic Supplementary Material

Table A1 Summary of the findings of a review study on factors affecting PhD students well-being, achievement and PhD completion; Table A2 Details of electronic survey; Table A3 Demographic characteristics of the participants; Table A4 Pearson’s correlations between all variables in the study; Table A5 Recommendations related to preventing frustration and supporting satisfaction of autonomy, competence and relatedness
