# Supplementary material for: Burnout and engagement among PhD students in medicine: the BEeP study
Source: Perspect Med Educ. 2020 Dec 7;10(2):110–7. doi: 10.1007/s40037-020-00637-6 (PMC7952475; doi:10.1007/s40037-020-00637-6)
Supplement: Supplementary file 1 — Table A1 Summary of the findings of a review study on factors affecting PhD students well-being, achievement and PhD completion; Table A2 Details of electronic survey; Table A3 Demographic characteristics of the participants; Table A4 Pearson’s correlations between all variables in the study; Table A5 Recommendations related to preventing frustration and supporting satisfaction of autonomy, competence and relatedness [file 40037_2020_637_MOESM1_ESM.docx]

**Appendix/ Electronic Supplementary information**

*Kusurkar, R.A., van der Burgt, S.M.E., Isik, U. et al. Burnout and engagement among PhD students in medicine: the BEeP study. Perspect Med Educ (2020).* [*https://doi.org/10.1007/s40037-020-00637-6*](https://doi.org/10.1007/s40037-020-00637-6)

**Table A1** Summary of the findings of a review study on factors affecting PhD students well-being, achievement and PhD completion ^1^

| **Type of Factors** | **Factors affecting PhD students well-being, achievement and PhD completion** | **Findings** |
| --- | --- | --- |
| ***External*** | Supervision | Most important and well-researched factor for PhD student satisfaction and well-being. Open, supportive and frequent communication from the supervisors is important for PhD student satisfaction. |
|  | Personal life | Lack of leisure is associated with PhD student depression, low well-being and burnout. Work life imbalance is the strongest predictor of psychological distress among PhD students. |
|  | Department structure and socialization within the department | Student socialization and opportunities and information provided by the department is important for PhD student success. An indicator of success rather than well-being. |
|  | Financial opportunities | Access to funding is associated with greater PhD student satisfaction and well-being. |
| ***Internal*** | Motivation | Intrinsic motivation is associated with PhD student satisfaction and well-being. |
|  | Academic writing skills and self-regulation | An indicator of success rather than well-being or satisfaction. |
|  | Academic Identity | Academic identity development through participation in formal activities, like conferences, and informal activities, like peer interaction, contributes to a positive PhD experience. The informal activities have more influence than the informal ones. Doesn’t say anything about well-being. |
|  | Self-worth | Fluctuations are seen in feelings of self-worth due to ongoing assessments and achievements or failures. An indicator of success rather than well-being. |
|  | Self-efficacy | Self-efficacy in research is associated with interest in research. An indicator of success rather than well-being. |

**Table A2** Details of electronic survey (75 items)

| **Sr. no.** | **Name of questionnaire/question (Description)** | **Variable measured and subscales** | **Total items** | **Likert scale** |
| --- | --- | --- | --- | --- |
|  | Maslach Burnout Inventory – SS (MBI-SS) ^2^ (Burnout is described as having three dimensions: exhaustion, cynicism and perceived negative efficacy.) | Burnout:   - Exhaustion - Cynicism - Perceived Negative Efficacy | 15 | 0-6 |
|  | Utrecht Work Engagement Scale (UWES-S-9)^2^ (Engagement is defined as “a positive, fulfilling, and work-related state of mind that is characterized by vigor, dedication and absorption”.^)^ | Engagement:   - Dedication - Vigour - Absorption | 9 | 0-4 |
|  | Academic Self-Regulation Questionnaire (SRQ-A)^3^ | Motivation:   - Autonomous Motivation - Controlled Motivation | 16 | 1-7 |
|  | Work-life balance scale^4^ (Work-life balance involves the perception of balance between work and personal life.) | Work-life balance | 7 | 1-3 |
|  | Do you feel like a part of a team at work? | Feeling of being a part of a team at work | 1 | 1-6 |
|  | Sleep^5^ (We chose two questions to measure the sleep quality. Quality of sleep is about having a good subjective quality of sleep in the night and feeling refreshed the next morning and is an indicator of well-being.) | - Subjective quality of sleep - Do you feeling refreshed after waking up? | 1  1 | 0-10  1-5 |
|  | Perceived conflict between different responsibilities at work (Perceived conflict in work-related responsibilities means the conflict that PhD students in medicine may feel between research work, clinical duties, teaching obligations and lab work.) | Perceived conflict between different responsibilities at work | 1 | 1-10 |
|  | Basic Psychological Needs Scale (BPNS) for measuring the score on satisfaction and frustration of autonomy, competence and relatedness at work.^6^ | - BPN Satisfaction - BPN Frustration | 24 | 1-7 |

**Table A3** Demographic characteristics of the participants

| **Variable** | **N** | **Range** | **Mean** | **Frequency distribution** | **Percentage** |
| --- | --- | --- | --- | --- | --- |
| Age | 460 | 21-66 years | 29.58 ± 5.33 | 21-24=16  25-34=406  35-66= 38 | 3.5%  88.2%  8.3% |
| Gender | 464 |  |  | Male=93  Female=371 | 20%  80% |
| Nationality | 464 |  |  | Dutch=390  International=61  Other=13 | 84.1%  13.1%  2.8% |
| Most recently completed degree | 439 |  |  | Bachelor Medicine=5  Bachelor Other=1  Master Medicine=146  Master Other=267  Medical specialist=9  PhD (completed)=11 | 1.1%  0.2%  33.3%  60.8%  2.1%  2.5% |
| Year of PhD | 172 |  |  | 1=34  2=48  3=41  4=36  5-8=13 | 19.8%  27.9%  23.8%  20.9%  7.6% |
| Marital status | 439 |  |  | Married=78  Widowed=1  Divorced=1  Single=91  In a relation=72  Live-in relation=196 | 17.8%  0.2%  0.2%  20.7%  16.4%  44.7% |
| Children | 435 |  |  | Yes=58  No=377 | 13.3%  86.7% |
| Department | 438 |  |  | Clinical=227  Non-clinical=196  Other=15 | 51.8%  44.8%  3.4% |
| Main work setting | 436 |  |  | Lab=117  With patients=74  Office=245 | 26.8%  17.0%  56.2% |
| Conflicted between work responsibilities | 439 | 1-10 | 4.7 ± 0.94 |  |  |
| Feel part of a team in your PhD work | 436 | 1-6 | 3.66 ± 1.53 |  |  |
| No. of PhD contract hours | 425 | 0-46 | 31.77 ± 10.18 | <36=100  36=294  >36=31 | 23.5%  69.2%  7.3% |
| No. of actual PhD hours per week | 407 | 0-75 |  | <36=89  36=40  >36=278 | 21.9%  9.8%  68.3% |
| Quality of sleep | 439 | 0-10 | 6.89 ± 1.75 |  |  |
| Do you feel refreshed when you wake up in the morning? | 428 | 1-5 | 3.17 ± 0.96 |  |  |

**Table A4** Pearson’s correlations between all variables in the study

| **Variables (Cronbach’s alpha for reliability)** | WLBS | Exh | Cynicism | Neg Per Self-eficacy | AM | CM | Vigour | Ded | Abs | AS | CS | RS | AF | CF | RF | BPNS sat | BPNS frus | Conflict | Team | Sleep | Refresh |
| --- | --- | --- | --- | --- | --- | --- | --- | --- | --- | --- | --- | --- | --- | --- | --- | --- | --- | --- | --- | --- | --- |
| WLBS (0.87) | 1 | - | - | - | - | - | - | - | - | - | - | - | - | - | - | - | - | - | - | - | - |
| Exhaustion (0.83) | -0.51* | 1 | - | - | - | - | - | - | - | - | - | - | - | - | - | - | - | - | - | - | - |
| Cynicism (0.88) | -0.23* | 0.67* | 1 | - | - | - | - | - | - | - | - | - | - | - | - | - | - | - | - | - | - |
| Perceived Negative Self-efficacy  (0.81) | -0.14* | 0.38* | 0.53* | 1 | - | - | - | - | - | - | - | - | - | - | - | - | - | - | - | - | - |
| Autonomous Motivation (0.84) | 0.07 | -0.36* | -0.47* | -0.52* | 1 | - | - | - | - | - | - | - | - | - | - | - | - | - | - | - | - |
| Controlled Motivation (0.82) | -0.13* | 0.35* | 0.41* | 0.34* | -0.22* | 1 | - | - | - | - | - | - | - | - | - | - | - | - | - | - | - |
| Vigour  (0.86) | 0.09 | -0.51* | -0.60* | -0.61* | 0.53* | -0.19* | 1 | - | - | - | - | - | - | - | - | - | - | - | - | - | - |
| Dedication (0.82) | 0.05 | -0.40* | -0.69* | -0.59* | 0.60* | -0.24* | 0.72* | 1 | - | - | - | - | - | - | - | - | - | - | - | - | - |
| Absorption (0.73) | -0.08 | -0.27* | -0.43* | -0.48* | 0.53* | -0.10* | 0.75* | 0.63* | 1 | - | - | - | - | - | - | - | - | - | - | - | - |
| Autonomy satisfaction (0.83) | 0.18* | -0.53* | -0.62* | -0.53* | 0.51* | -0.18* | 0.65* | 0.63* | 0.51* | 1 | - | - | - | - | - | - | - | - | - | - | - |
| Competence satisfaction (0.90) | 0.24* | -0.44* | -0.46* | -0.69* | 0.32* | -0.32* | 0.55* | 0.45* | 0.37* | 0.52* | 1 | - | - | - | - | - | - | - | - | - | - |
| Relatedness satisfaction (0.87) | 0.19* | -0.32* | -0.29* | -0.26* | 0.21* | -0.19* | 0.27* | 0.24* | 0.20* | 0.35* | 0.35* | 1 | - | - | - | - | - | - | - | - | - |
| Autonomy frustration (0.79) | -0.43* | 0.67* | 0.62* | 0.38* | -0.40* | 0.33* | -0.47* | -0.45* | -0.29* | -0.61* | -0.38* | -0.25* | 1 | - | - | - | - | - | - | - | - |
| Competence frustration (0.86) | -0.27* | 0.50* | 0.48* | 0.59* | -0.22* | 0.41* | -0.46* | -0.34* | -0.27* | -0.39* | -0.75* | -0.27* | 0.44* | 1 | - | - | - | - | - | - | - |
| Relatedness frustration (0.84) | -0.25* | 0.36* | 0.35* | 0.23* | -0.10^!^ | 0.33* | -0.25* | -0.20* | -0.17* | -0.24* | -0.34* | -0.76* | 0.33* | 0.39* | 1 | - | - | - | - | - | - |
| BPNS satisfaction (0.88) | 0.26* | -0.55* | -0.58* | -0.63* | 0.44* | -0.29* | 0.63* | 0.56* | 0.46* | 0.80* | 0.79* | 0.74* | -0.53* | -0.60* | -0.58* | 1 | - | - | - | - | - |
| BPNS frustration (0.86) | -0.41* | 0.66* | 0.63* | 0.53* | -0.31* | 0.46* | -0.51* | -0.43* | -0.32* | -0.54* | -0.65* | -0.54* | 0.75* | 0.81* | 0.73* | -0.74 | 1 | - | - | - | - |
| Work responsibility conflict | -0.42* | 0.34* | 0.22* | 0.17* | -0.10^!^ | 0.19* | -0.06 | -0.02 | 0.08 | -0.15* | -0.20* | -0.10^!^ | 0.41* | 0.21* | 0.09 | -0.19* | 0.30* | 1 | - | - | - |
| Part of a team | 0.15* | -0.30* | -0.33* | -0.29* | 0.24* | -0.16* | 0.26* | 0.30* | 0.18* | 0.27* | 0.21* | 0.40* | -0.20* | -0.14* | -0.36* | 0.38* | -0.29* | -0.06 | 1 | - | - |
| Quality of sleep | 0.38* | -0.39* | -0.20* | -0.13* | 0.14* | -0.80 | 0.20* | 0.11* | 0.09 | 0.19* | 0.12* | 0.19* | -0.26* | -0.21* | -0.21* | 0.22* | -.0.29* | -0.16* | 0.12* | 1 | - |
| Refreshed in the morning | 0.31* | -0.45* | -0.32* | -0.26* | 0.23* | -0.11^!^ | 0.35* | 0.26* | 0.19* | 0.29* | 0.28* | 0.14* | -0.31* | -0.32* | -0.16* | 0.30* | -0.34* | -0.17* | 0.15* | 0.58* | 1 |
| Discrepancy in working hours | 0.07 | 0.06 | 0.06 | 0.04 | -0.01 | -0.13^!^ | 0.12^!^ | -0.02 | 0.16* | -0.08 | 0.10^!^ | -0.13^!^ | 0.12^!^ | -0.02 | 0.16* | -0.08 | 0.10^!^ | -0.03 | 0.01 | -0.11^!^ | -0.06 |

**p<0.001, ^!^p<0.05*

**Table A5** Recommendations related to preventing frustration and supporting satisfaction of autonomy, competence and relatedness

| **PhD students** | **Supervisors** | **Organizations** |
| --- | --- | --- |
| If your basic psychological needs are getting frustrated, try to change the situation. ***(A, C ,R)*** | Get training for supervisory skills as an addition to your research skills repertoire. ***(C)*** | Organize courses for PhD supervisors on supervision skills, which are obligatory. ***(C)*** |
| Reinstate autonomy in your work and initiate conversations with your supervisors if needed. ***(A)*** | Follow training courses specifically on feedback. ***(C)*** | Provide feedback training for supervisors. ***(C)*** |
| Break down challenging tasks into smaller steps of competence. ***(C)*** | Take half yearly/yearly feedback from your PhD students. ***(C, R)*** | Provide for sharing supervision expertise and experiences. ***(C, R)*** |
| Build a strong community of PhD students to discuss your experiences and problems, and to share failures and successes. ***(R)*** | Stimulate a culture of safety, openness and warmth in your department. ***(R)*** | Make feedback on supervision skills a part of performance appraisals. (***C)*** |
| **Covey’s circle of concern and circle of influence^7^** – Focus on the circle of influence because these are things that you can change yourself. For things that lie in the circle of concern, recruit the help of seniors, peers, co-supervisors, etc. to bring about changes in your situation. ***(A, C, R)***  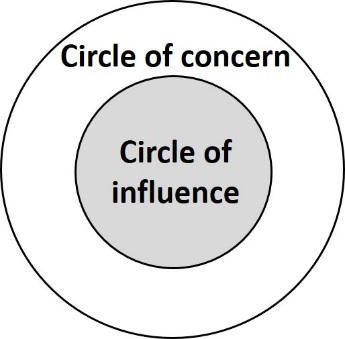 | Participate in regular informal sessions with your PhD students. ***(C, R)*** | Organize psychological support for PhD students, advertise it and keep it visible so that the students can find and approach it easily. ***(R)*** |
| Celebrate successes with peers and supervision team. ***(C, R)*** | Celebrate successes of PhD students and give compliments. ***(C, R)*** |  |

*A - Autonomy, C- Competence, R – Relatedness*

*These recommendations are based on our findings and Self-determination Theory.*

**Figure A1 Cluster profiles as they score on the dependent variables**


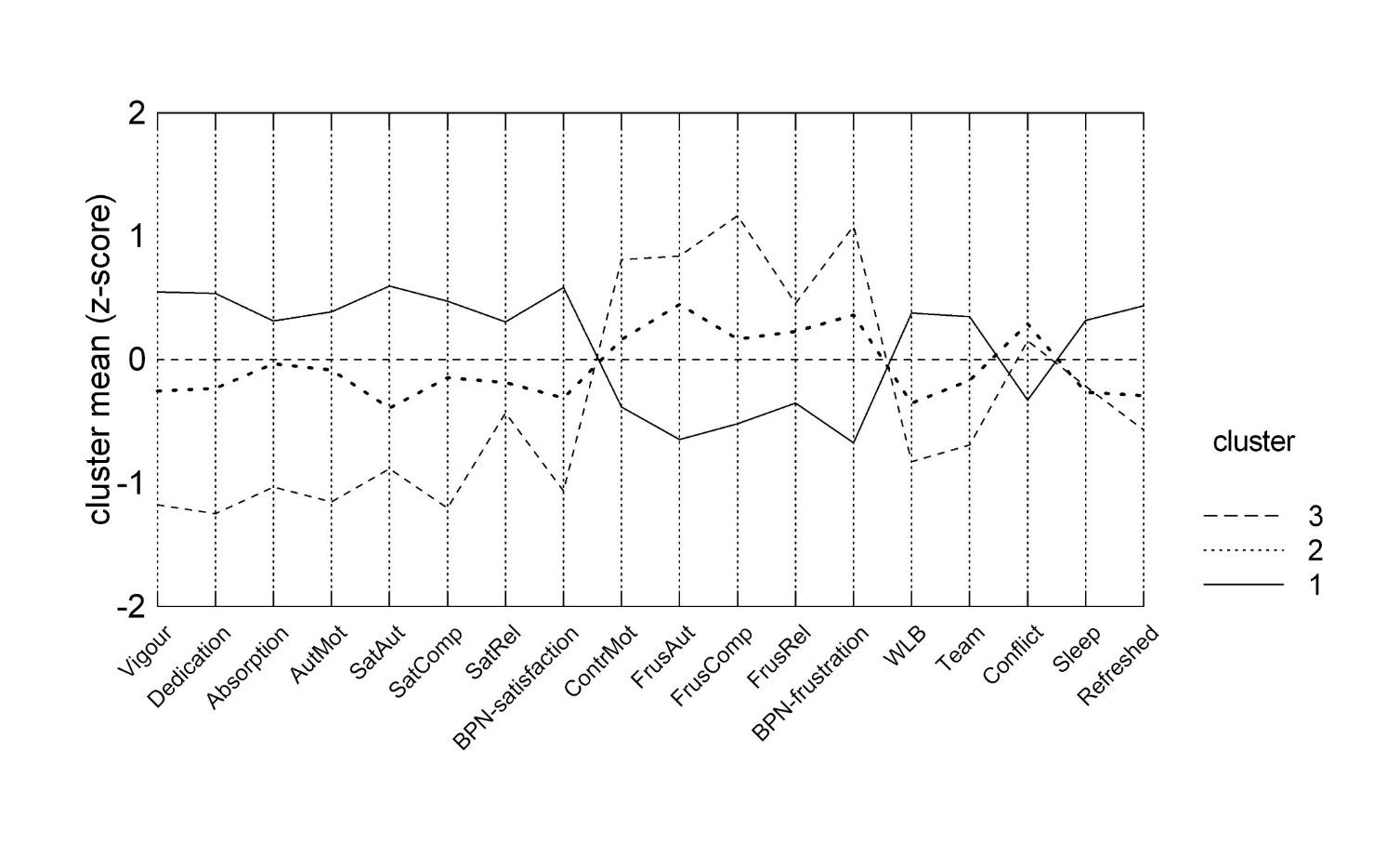


**Acknowledgements**

This research was conducted at the request of the PhD-student Representative Committee (Pro-VUmc) of Amsterdam UMC, location VUmc. We worked together with them to finalize the survey, mail it to the PhD students and increase the response rate. They also played a major role in procuring funding for this study from the Amsterdam UMC Research Institutes.

We would like to thank Pro-VUmc members (Heder de Vries, Nicole den Braver, Wieke Kremer, Marieke Heineke, Kimmy Rosielle, David Cucchi, Marta Lopez Gonzalez, Ivy van Dijke, Sophie Horrevorts, Ludo Haasterecht, Floris van den Brand and Najada Stringa) for their staunch support and collaboration in making this project a success, especially for using the study results to make concrete recommendations to the Board of Directors of the hospital and the Amsterdam Research Board on improving PhD students’ working environment. We would like to thank the Board of Directors of the hospital, for their support on this research and willingness to take up the recommendations. We would like to thank the Research Institutes of Amsterdam UMC and the HR department for the funding they provided for this study. We would also like to thank our Research Assistant, Bart van Elswijk, Amsterdam UMC-location VUmc, for setting up the electronic questionnaire and all his help with the study.

**References**

1. Sverdlik A, Hall NC, McAlpine L, Hubbard K. The PhD experience: A review of the factors influencing doctoral students’ completion, achievement, and well-being. Int J Doct Stud. 2018;13:362-87.
2. Schaufeli WB, Martinez IM, Pinto AM, Salanova M, Bakker AB. Burnout and engagement in university students: A cross-national study. J Cross Cult Psych 2002;33:464–81.
3. Vansteenkiste M, Zhou M, Lens W, Soenens B. Experiences of autonomy and control among Chinese learners: Vitalizing or Immobilizing? J Educ Psychol. 2005;97:468–83.
4. Dex S, Bond S. Measuring work-life balance and its covariates. Work Employ Soc. 2005;19:627-37.
5. Campbell R, Tobback E, Delesie L, Vogelaers D, Mariman A, Vansteenkiste M. Basic psychological need experiences, fatigue, and sleep in individuals with unexplained chronic fatigue. Stress Health. 2017;33:645-55.
6. Chen B, Vansteenkiste M, Beyers W, et al. Basic psychological need satisfaction, need frustration, and need strength across four cultures. Mot Emot. 2015;39:216-36.
7. Covey SR. Principle-centered leadership. NY: Free Press 1991.
